# Supplementary material for: Genetic Architecture Underlying the Metabolites of Chlorogenic Acid Biosynthesis in Populus tomentosa
Source: Int J Mol Sci. 2021 Feb 27;22(5):2386. doi: 10.3390/ijms22052386 (PMC7957499; doi:10.3390/ijms22052386)
Supplement: Supplementary file 1 [file ijms-22-02386-s001.zip › Supplementary Information.pdf]

Genetic Architecture Underlying the Metabolites of Chlorogenic Acid Biosynthesis in *Populus tomentosa*

Liangchen Yao [1,2,†], Peng Li [1,2,†], Qingzhang Du [1,2,3], Mingyang Quan [1,2], Lianzheng Li [1,2], Liang Xiao [1,2], Fangyuan Song [1,2], Wenjie Lu [1,2], Yuanyuan Fang [1,2] and Deqiang Zhang [1,2,*]

[1] National Engineering Laboratory for Tree Breeding, College of Biological Sciences and Technology, Beijing Forestry University, No. 35, Qinghua East Road, Beijing 100083, China; liangchenyao@bjfu.edu.cn (L.Y.); lipeng@bjfu.edu.cn (P.L.); Qingzhangdu@bjfu.edu.cn (Q.D.); Mingyangquan@bjfu.edu.cn (M.Q.); lzlee@bjfu.edu.cn (L.L.); xiaoliang0622@126.com (L.X.); fangyuansong@bjfu.edu.cn (F.S.); wenjielu@bjfu.edu.cn (W.L.); yuanyuanfang@bjfu.edu.cn (Y.F.)

[2] Key Laboratory of Genetics and Breeding in Forest Trees and Ornamental Plants, Ministry of Education, College of Biological Sciences and Technology, Beijing Forestry University, No. 35, Qinghua East Road, Beijing 100083, China

[3] Beijing Advanced Innovation Center for Tree Breeding by Molecular Design, College of Biological Sciences and Technology, Beijing Forestry University, No. 35, Qinghua East Road, Beijing 100083, China

* Correspondence: DeqiangZhang@bjfu.edu.cn; Tel.: +86-10-62336007; Fax: +86-10-62336164

† These authors contributed equally to this work.

The following Supplementary Information is available for this article:

Method S1. Sample treatment for metabolite analysis.

Method S2. RNA extraction and RNA sequencing.

Supplementary Figures

Figure S1. Subpopulation differentiation patterns of six metabolites involved in CGA biosynthesis.

Figure S2. Manhattan graphs of eight metabolites involved in CGA biosynthesis.

Figure S3. SNP18_9299320 and the associated *HCT* are located within a 5 kb LD block.

Figure S4. Possible transcriptional regulatory network between genes under drought stress.

Supplementary Tables

Table S1. Statistical analysis of 11 metabolic traits involved in CGA biosynthesis in *P. tomentosa*.

Table S2. Analysis of phenotypic variation in the different subpopulations.

Table S3. Multiple comparisons of phenotypic data from the different subpopulations.

Table S4. Full details of the genome-wide association studies (GWAS) of 11 metabolites involved in CGA biosynthesis among *P. tomentosa*.

Table S5. mGWAS and eQTN-associated genes located in the selective sweep region of the genome.

Table S6. Gene Ontology (GO) enrichment analysis of all genes detected by mGWAS and eQTN mapping (FDR ≤ 0.05).

Table S7. Details of the association analysis of candidate genes and target traits in *P. tomentosa*.

Table S8. Correlation between causal gene expression levels and metabolite abundance.

Supplemental Data

Data S1-S3 were provided separately as an Excel file.

Data S1. Detailed information listing the annotation of causal genes in GWAS and the best BLAST results for *Populus trichocarpa* and *Arabidopsis thaliana*.

Data S2. Detailed information listing the eQTNs identified for each gene in the mGWAS.

Data S3. Detailed information listing the annotation of causal genes in the eQTN mapping and the best BLAST results for *Populus trichocarpa* and *Arabidopsis thaliana*.

Method S1. Sample treatment for metabolite analysis

We used LC-electrospray ionization (ESI)-MS/MS system (HPLC, Shim-pack UFLC SHIMADZU CBM30A system; MS, Applied Biosystems 6500 Q TRAP) to analyze the sample extraction. The analysis conditions are set as follows, HPLC: column, Waters ACQUITY UPLC HSS T3 C18 (1.8 µm, 2.1 mm*100 mm); solvent system, water (0.04% acetic acid): acetonitrile (0.04% acetic acid); gradient program,100:0V/V at 0 min, 5:95V/V at 11.0 min, 5:95V/V at 12.0 min, 95:5V/V at 12.1 min, 95:5V/V at 15.0 min; flow rate, 0.40 ml/min; temperature, 40°C; injection volume: 2 μl. The effluent was alternatively connected to an ESI-triple quadrupole-linear ion trap (QTRAP)-MS. Using the triple quadrupole linear ion trap mass spectrometer (Q TRAP), API 6500 Q TRAP LC/MS/MS system to capture LIT and triple quadrupole (QQQ) scanning, the system is controlled by Analyst 1.6 software (AB Sciex), it equipped with ESI Turbo Ion-Spray interface and runs in positive ion mode. The ESI source operating parameters are set as follows: ion source, turbo spray; source temperature 500°C; ion spray voltage (IS) 5500 V; ion source gas I (GSI), gas II (GSII), curtain gas (CUR) was set at 5560, and 25.0 psi, respectively; the collision gas (CAD) was high. The instrument calibration and mass calibration were performed in QQ and LIT modes using 10 and 100 μmol/L polypropylene glycol solutions, respectively. The QQQ scan was obtained in the MRM experiment with the collision gas (nitrogen) set to 5 psi. Further through DP and CE optimization, the DP and CE of an individual MRM conversions are completed. Based on the metabolites eluted during this period, a specific set of monitoring is performed on the MRM transition at each period.

Method S2. RNA extraction and RNA sequencing

Total RNA from fresh leaves samples was extracted by a Qiagen RNeasy kit (Qiagen China, Shanghai, China) following to the manufacturer's protocols. In addition, DNase digestion was executed when RNA was purified using RNase-free DNase set (Qiagen). RNA samples were evaluated using NanoDrop ND-1000 (A260/A280 = 1.91) and Agilent Bioanalyzer 2100 (28S/18S = 1.6). According to the manufacturer's recommendations and index codes, we use the NEBNext® UltraTM RNA Library Prep

Kit for Illumina® (NEB, USA), and a total of 3 μg RNA per sample is constructed a strand-specific RNA-seq library. Cluster the index-coded samples using TruSeq PE Cluster Kit v3-cBot-HS (Illumia) on the cBot Cluster Generation System abide by the manufacturer's instructions. The pair sequencing was performed on the Illumina HiSeq 2500 platform (Illumina) to generate 100-nt paired-end reads with the user guide. we download the original data from the NCBI Sequence Read Archive. To obtain high-quality data, the reads containing adapter, reads containing ploy-N and low-quality reads were removed from original data by in-house perl scripts. Then, high-quality data was uniquely mapped to the *Populus tomentosa* reference genome using TopHat 2.1.1 with default options (Trapnell *et al*., 2009). Based on fragments per kilobase of transcript per million fragments (FPKM) values, the isoform level and gene level counts of the assembled transcripts are calculated and standardized by using Cufflinks v2.1.1 (Trapnell *et al*., 2012) with default options.

References:

Trapnell, C.; Pachter L.; Salzberg S.L. TopHat: discovering splice junctions with RNA-Seq. *Bioinformatics*. **2009**, 25, 1105-11. DOI: 10.1093/bioinformatics/btp120.

Trapnell, C.; Roberts, A.; Goff, L.; Pertea, G.; Kim, D.; Kelley, D.R.; Pimentel, H.; Salzberg, S.L.; Rinn J.L.; Pachter, L. Differential gene and transcript expression analysis of RNA-seq experiments with TopHat and Cufflinks. *Nat Protoc*. **2012**, 7, 562-78. DOI: 10.1038/nprot.2012.016.

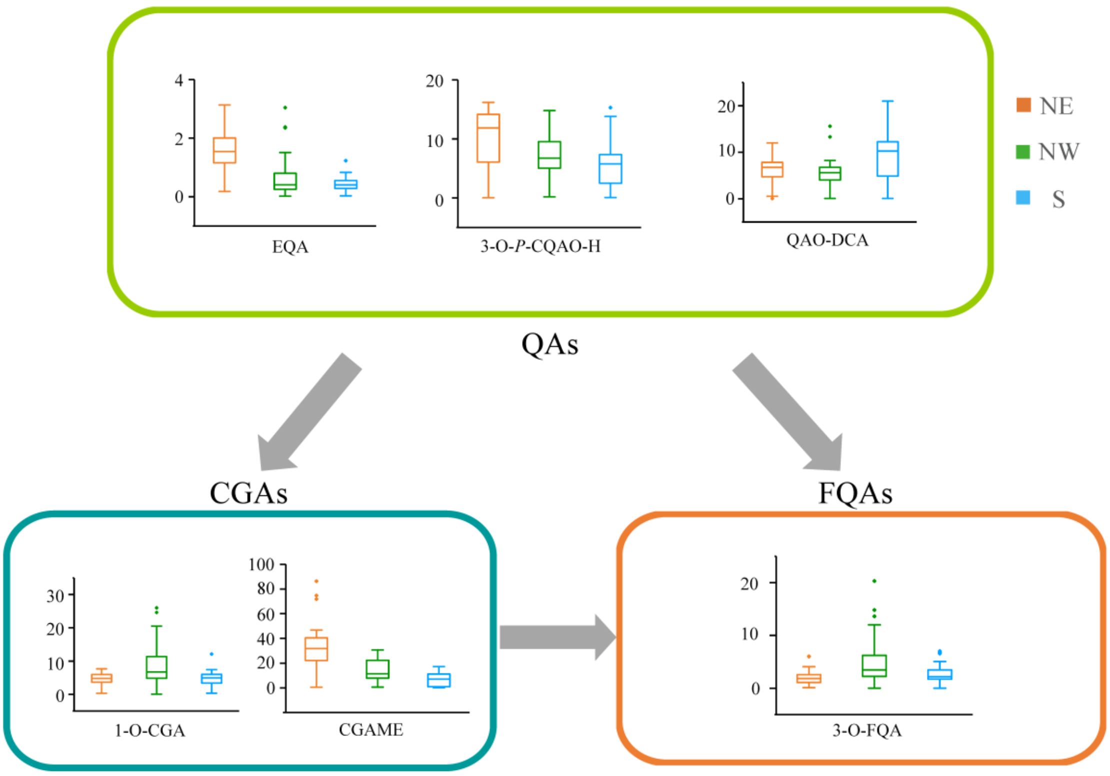

Figure S1. Subpopulation differentiation patterns of six metabolites involved in CGA biosynthesis.

Three square boxes of various colors represent the remaining three types of metabolites, and the gray arrows indicate the transformation relationship. QAs include eudesmoyl quinic acid (EQA); 3-O-*p*-coumaroyl quinic acid O-hexoside (3-O-*P*-CQAO-H); quinic acid O-di-glucuronic acid (QAO-DCA), CGAs include 1-O-chlorogenic acid (1-O-CGA); chlorogenic acid methyl ester (CGAME), and 3-O-feruloylquinic acid (3-O-FQA) corresponds to FQAs. The box plots show the phenotypic differentiation of six metabolites related to CGA biosynthesis, which ordinate values were shrunk by 10^5, simultaneously. Three sub-populations corresponding to southern (S), northwestern (NW), and northeastern (NE) regions of China.

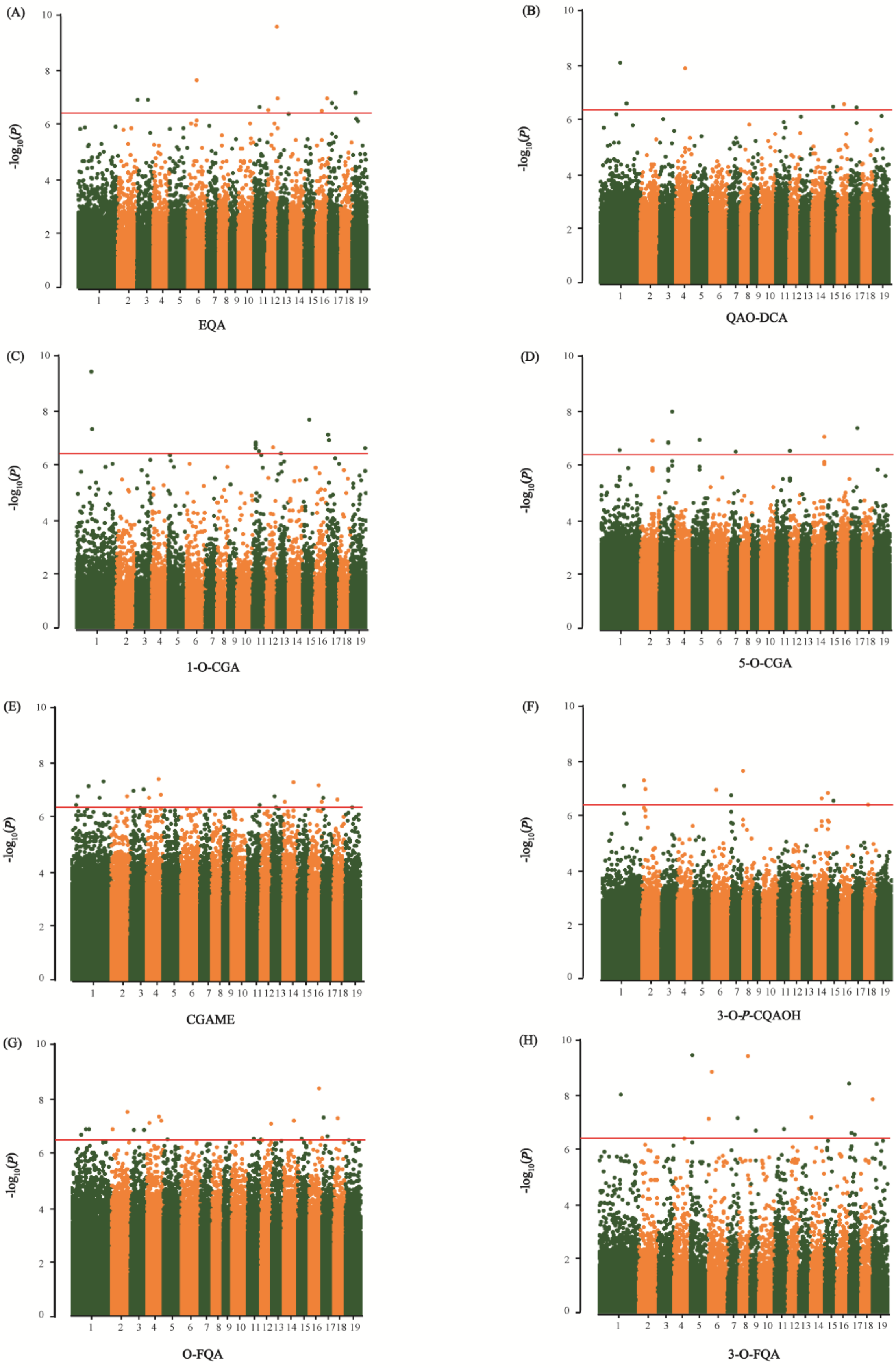

Figure S2. Manhattan graphs of eight metabolites involved in CGA biosynthesis.

The red line in each Manhattan plot represents the Bonferroni-adjusted significance

threshold ($P \leq 4.14E\text{-}07$). The *x* and *y* axes show genomic positions, denoted as -log10 (*p*), respectively. Besides, 5-O-CGA and O-FQA indicate 5-O-chlorogenic acid and O-feruloylquinic acid, respectively.

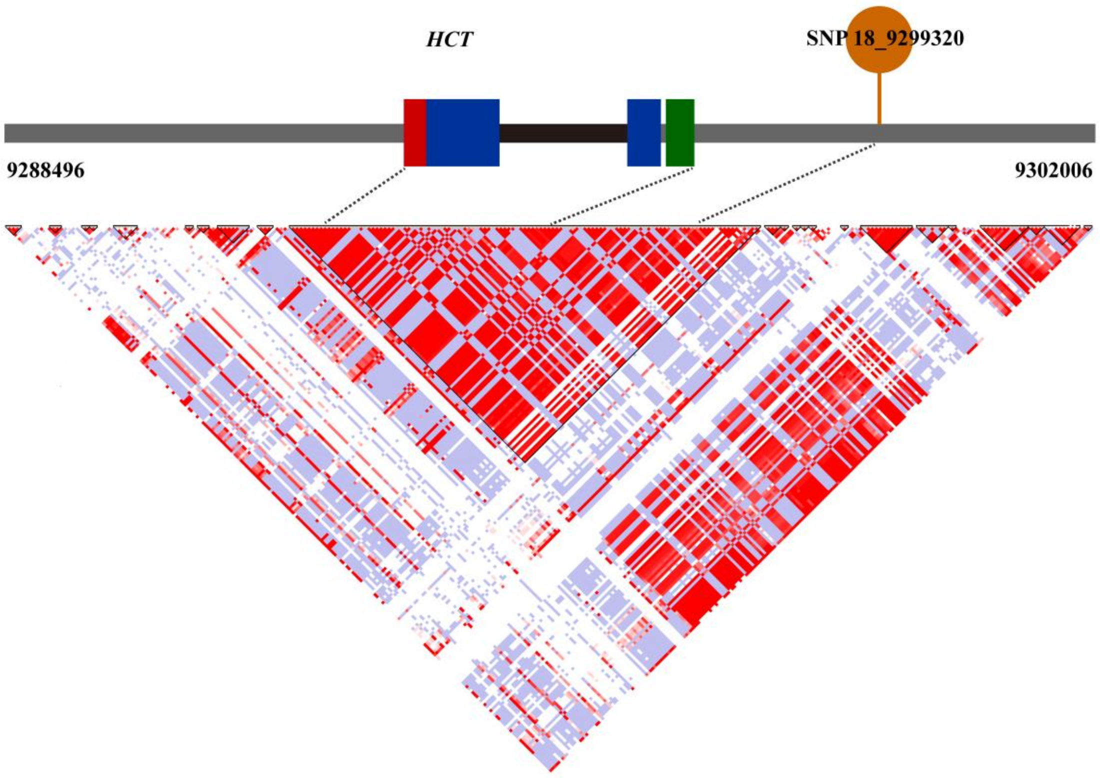

Figure S3. SNP18_9299320 and the associated *HCT* are located within a 5 kb LD block.

LD block is displayed in *Ptom.018G.00976* (*HCT*) and its flanking sequence. The structure of *HCT* is shown at the top (red, 5'-UTR; blue, exon; green, 3'-UTR; black, Intron; Grey, flanking sequence of gene), and the position of the association locus SNP18_9299320 on the chromosome is also marked. The nether picture was a linkage disequilibrium (LD) representation of the pair-wise r^2 value among all polymorphic sites across a 5-kb flanking region of *HCT*, and the redness of the box is proportional to the r^2 value.

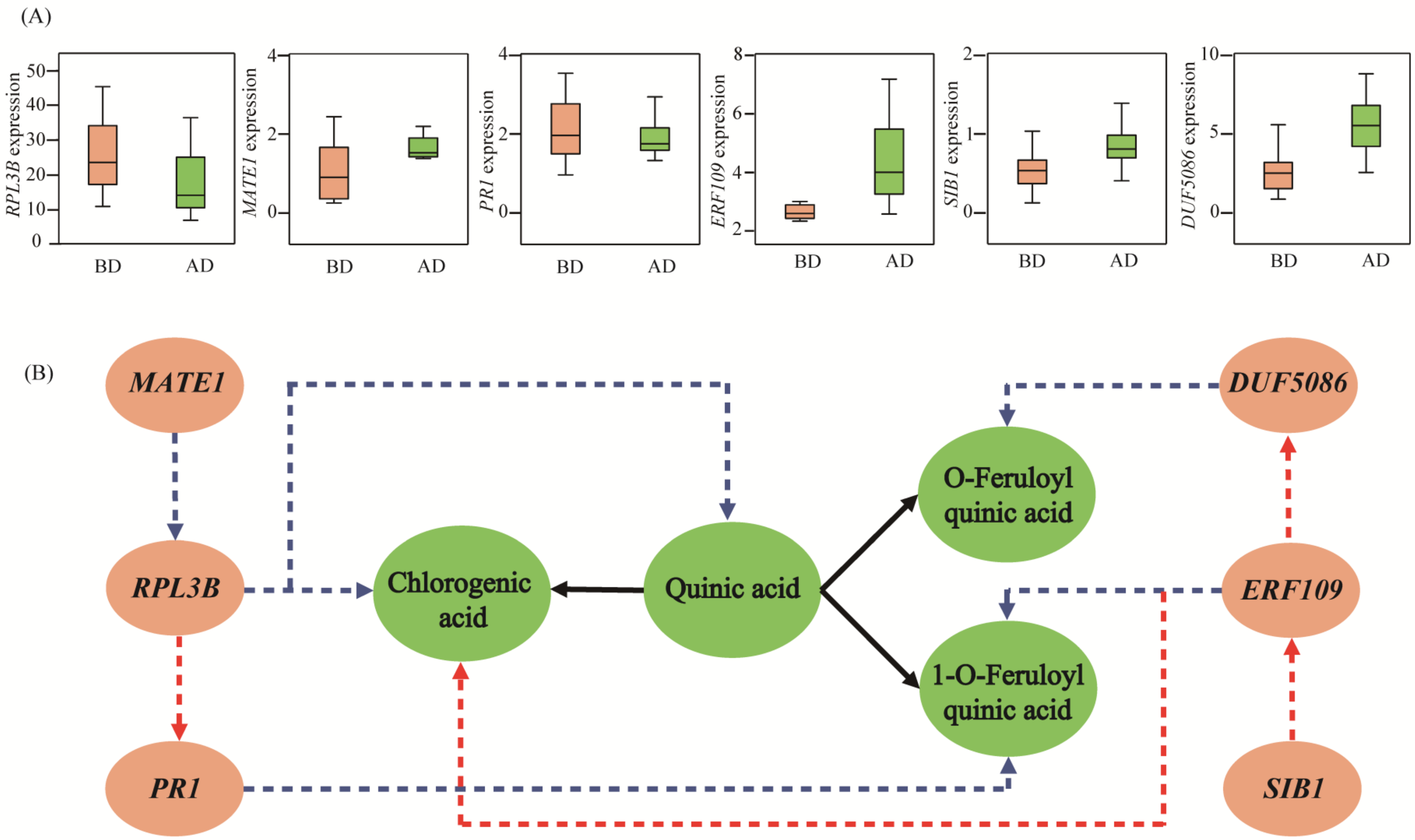
(A)
RPL3B expression
MATE1 expression
PR1 expression
ERF109 expression
SIB1 expression
DUF5086 expression
BD
AD
(B)
MATE1
RPL3B
PR1
DUF5086
ERF109
SIB1
Chlorogenic acid
Quinic acid
O-Feruloyl quinic acid
1-O-Feruloyl quinic acid

Figure S4. Possible transcriptional regulatory network between genes under drought stress.

(A) The box plots show the expression transformative mode of six causal genes before and after drought resistance. *RPL3B*, *Ptom.003G.01892*; *MATE1*, *Ptom.010G.02355*; *PR1*, *Ptom.006G.00814*; *ERF109*, *Ptom.009G.00855*; *SIB1*, *Ptom.001G.00290*; *DUF5086*, *Ptom.001G.00737*. BD and AD represent the expression abundance before and after drought, respectively. (B) The green and red circles represent metabolites and genes, respectively. The black line represents the conversion relationship between metabolites, and the red and blue dashed lines represent significant positive and negative correlations, respectively.

Table S1. Statistical analysis of 11 metabolic traits involved in CGA biosynthesis in *P. tomentosa*

Metabolite traits[a]	Max	Min	Average	SD[b]	SK[c]	CV[d]
QA	1250000	100	294531	215441.7326	1.691994236	0.731473877
EQA	313000	2000	83035	77689.14789	1.316831677	0.935615308
QAO-DCA	2100000	3000	680790	436771.6862	0.502264422	0.641565955
1-O-CGA	2600000	7000	593566	481594.6819	2.007239887	0.811358738
3-O-CGA	187000	200	118612	53514.61535	-1.15016197	0.451173704
5-O-CGA	87400	2500	33766	23082.99995	0.335277143	0.683610043
CGAME	8630000	4000	1635900	1597034.968	1.909663005	0.976242416
3-O-*P*-CGAO-H	1620000	2000	728300	466429.2336	0.134783731	0.640435581
O-FQA	7630000	6000	1593606	1437482.773	1.410346043	0.902031442
1-O-FQA	9770000	30000	3505200	2021802.898	-0.023086029	0.576801009
3-O-FQA	2030000	700	326422	324315.7716	2.642992576	0.993546853

[a]The metabolites are quinic acid, eudesmoyl quinic acid, quinic acid O-di-glucuronic acid, 1-O-chlorogenic acid, 3-O-chlorogenic acid, 5-O-chlorogenic acid, chlorogenic acid methyl ester, 3-O-*p*-coumaroyl quinic acid O-hexoside, O-feruloylquinic acid, 1-O-feruloylquinic acid, 3-O-feruloylquinic acid in order.

[b]Standard deviation

[c]Skewedness

[d]Coefficient of variation

Table S2. Analysis of phenotypic variation in the different subpopulations

Metabolite traits	Group mean		Sum of squares between groups	Within groups sum of squares	F^a	P^b
QA	NE	208289.2857	1.58592E+12	7.92958E+11	25.17247077	1.56376E-09
	NW	508638.8889				
	S	287250				
EQA	NE	176178.5714	2.43331E+11	1.21665E+11	32.97590242	1.25459E-11
	NW	72194.44444				
	S	48100.25				
QAO-DCA	NE	763321.4286	2.27911E+12	1.13955E+12	6.580405017	0.002089843
	NW	668611.1111				
	S	1057777.778				
1-O-CGA	NE	527000	3.97423E+12	1.98711E+12	10.04692945	0.000109188
	NW	1137611.111				
	S	592805.8056				
3-O-CGA	NE	110478.5714	26176165917	13088082959	4.879010309	0.009572034
	NW	168638.8889				
	S	138436.1111				
5-O-CGA	NE	27832.14286	5624377806	2812188903	5.733930761	0.00445647
	NW	46786.36111				
	S	47561.36111				
CGAME	NE	3524000	1.02311E+14	5.11553E+13	32.48663593	1.58645E-11
	NW	1616861.111				
	S	917166.6667				
3-O-*P*-CQAO-H	NE	1166750	3.02424E+12	1.51212E+12	7.830481842	0.000703746
	NW	821305.5556				
	S	719777.7778				
O-FQA	NE	3760107.143	7.00562E+13	3.50281E+13	24.99904753	1.82119E-09

	NW	1728583.333				
	S	1009305.806				
	NE	5677500				
1-O-FQA	NW	4730000	3.83976E+13	1.91988E+13	5.028156323	0.008360524
	S	3473333.333				
	NE	232646.4286				
3-O-FQA	NW	600388.8889	1.59062E+12	7.95311E+11	8.65422398	0.000350291
	S	277825.25				

[a]Ratio of squared deviation to degree of freedom between groups and groups.

[b]The level of significance.

Data from one-way analysis of variance.

Table S3. Multiple comparisons of phenotypic data from the different subpopulations

Metabolite traits	Subpopulation comparison	Mean difference	Standard error	Statistical significance
QA	NE-NW	-297242.46032*	44722.07428	1.76085E-09
	NE-S	-75353.57143	44722.07428	0.095216924
	NW-S	221888.88889*	41833.66989	7.13443E-07
EQA	NE-NW	94962.69841*	15259.32319	1.23934E-08
	NE-S	121431.89286*	15259.32319	3.29723E-12
	NW-S	26469.19444	14273.78983	0.066721292
QAO-DCA	NE-NW	50781.74603	104857.775	0.629270588
	NE-S	-283134.92063*	104857.775	0.00817816
	NW-S	-333916.66667*	98085.46708	0.000965764
1-O-CGA	NE-NW	-414861.11111*	112035.0221	0.000354079
	NE-S	15194.19444	112035.0221	0.892402707
	NW-S	430055.30556*	104799.167	8.47626E-05
3-O-CGA	NE-NW	-40481.74603*	13050.64188	0.002518444
	NE-S	-26753.96825*	13050.64188	0.043061657
	NW-S	13727.77778	12207.75765	0.263570646
5-OCGA	NE-NW	-15572.07540*	5691.108371	0.007392118
	NE-S	-15822.07540*	5691.108371	0.006527151
	NW-S	-250	5323.544418	0.962640708
CGAME	NE-NW	1828567.46032*	316193.1585	8.94218E-08
	NE-S	2496511.90476*	316193.1585	4.47089E-12
	NW-S	667944.44444*	295771.6168	0.026166591
3-O-*P*-CGAO-H	NE-NW	283015.87302*	110728.3306	0.012142409
	NE-S	435793.65079*	110728.3306	0.000156057

	NW-S	152777.7778	103576.8690	0.143446129
	NE-NW	1662452.38095*	297581.4359	2.11755E-07
O-FQA	NE-S	2042729.90873*	297581.4359	6.33586E-10
	NW-S	380277.5278	278361.9444	0.175058262
	NE-NW	461071.4286	492370.9203	0.351376898
1-O-FQA	NE-S	1492738.09524*	492370.9203	0.003118469
	NW-S	1031666.66667*	460570.8227	0.027375404
	NE-NW	-296456.74603*	76284.06375	0.000186241
3-O-FQA	NE-S	-63893.10714	76284.06375	0.404332222
	NW-S	232563.63889*	71357.20765	0.001541506

* represent a significant difference in the mean value of 0.001.

Back Testing with Least-SignificantDifference (LSD).

Table S4. Full details of the genome-wide association studies (GWAS) of 11 metabolites involved in CGA biosynthesis among *P. tomentosa*

Trait	SNP_ID	Add_effects	Dom_effects	*P*-value	R^2 (%)	*Populus tomentosa* gene
QA	Chr6_10068379			1.074E-07	10.18	Ptom.006G.01198
QA	Chr6_12256106	1.01	-0.05	1.905E-07	5.66	Ptom.006G.01409
QA	Chr6_12521430			1.262E-07	12.16	Ptom.006G.01429
QA	Chr6_14447761			1.644E-07	13.11	
QA	Chr6_15267207			1.674E-07	10.16	Ptom.006G.01609; Ptom.006G.01608; Ptom.006G.01610
QA	Chr6_15880648			1.351E-07	9.93	Ptom.006G.01662
QA	Chr10_3574428			1.14E-07	9.81	Ptom.010G.00539; Ptom.010G.00538
QA	Chr10_5499416			1.564E-07	9.84	Ptom.010G.00832; Ptom.010G.00834; Ptom.010G.00833 Ptom.010G.00836; Ptom.010G.00835
QA	Chr10_15191485			1.144E-07	9.71	
QA	Chr10_18270573			1.477E-07	9.71	Ptom.010G.02294; Ptom.010G.02293
QA	Chr1_6787904			1.524E-08	8.48	Ptom.001G.00759
QA	Chr1_9835177			1.479E-07	9.59	Ptom.001G.01099; Ptom.001G.01100
QA	Chr1_22551411			1.674E-07	10.16	
QA	Chr1_50281019			1.674E-07	10.16	Ptom.001G.04203
QA	Chr5_4679088	2.16	-0.23	2.883E-07	12.73	
QA	Chr5_6215994	1.05	-0.13	1.905E-07	5.77	Ptom.005G.00810; Ptom.005G.00811; Ptom.005G.00813
QA	Chr5_6284863			1.637E-07	9.63	Ptom.005G.00818
QA	Chr5_15529010			1.337E-07	9.72	Ptom.005G.01592
QA	Chr4_892692			1.134E-07	15.56	Ptom.004G.00111
QA	Chr4_17884540			1.674E-07	10.16	Ptom.004G.01782; Ptom.004G.01783
QA	Chr4_18831443	1.20	0.07	5.645E-08	10.82	Ptom.004G.01883
QA	Chr4_19073089			4.918E-08	4.37	Ptom.004G.01913

QA	Chr3_9709791	1.37	-0.47	8.114E-09	11.67	Ptom.003G.01253; Ptom.003G.01254
QA	Chr3_13723884			1.397E-07	10.09	
QA	Chr3_18929877	1.29	-0.40	3.78E-08	10.49	Ptom.003G.01892
QA	Chr11_12498534			1.077E-07	12.10	Ptom.011G.00785
QA	Chr15_3079190			1.674E-07	10.16	
QA	Chr15_10233479	1.29	-0.42	3.78E-07	10.55	Ptom.015G.00853; Ptom.015G.00851; Ptom.015G.00850
QA	Chr17_10693566	0.92	-0.26	2.419E-07	5.67	
QA	Chr2_13241941			1.651E-07	10.10	Ptom.002G.01847; Ptom.002G.01846
QA	Chr2_15106543			1.617E-07	9.60	
QA	Chr2_17659619			1.161E-07	9.72	
QA	Chr7_2712964	2.16	-0.23	2.883E-07	12.73	Ptom.007G.00352; Ptom.007G.00353
QA	Chr7_12829919			1.277E-07	9.99	Ptom.007G.01300; Ptom.007G.01301
QA	Chr14_10437267			1.298E-07	9.76	Ptom.014G.01270
QA	Chr19_14925415	1.66	-0.71	1.469E-07	30.25	
QA	Chr19_14077575			3.834E-07	9.34	
EQA	Chr6_11283146			2.503E-08	30.88	Ptom.006G.01326; Ptom.006G.01325
EQA	Chr3_742651			1.304E-07	29.53	Ptom.003G.00117; Ptom.003G.00118
EQA	Chr3_13434284			1.304E-07	29.53	
EQA	Chr11_6844231			2.447E-07	28.00	
EQA	Chr16_6988048			3.4E-07	25.72	Ptom.016G.00646
EQA	Chr16_13932040	1.33	-0.53	1.15E-07	21.30	
EQA	Chr17_3997334			1.765E-07	27.03	Ptom.017G.00416
EQA	Chr17_8716618			2.557E-07	25.73	
EQA	Chr12_415522			3.049E-07	28.11	
EQA	Chr12_11545572	1.37	0.13	2.855E-10	29.10	
EQA	Chr12_12306425			1.18E-07	35.35	Ptom.012G.01133; Ptom.012G.01131

EQA	Chr19_3938704			7.471E-08	29.85	
QAO-DCA	Chr1_24681847			7.563E-09	5.39	
QAO-DCA	Chr1_32796743			2.35E-07	3.65	
QAO-DCA	Chr4_12174740			1.196E-08	3.15	Ptom.004G.01213; Ptom.004G.01214
QAO-DCA	Chr3_6370190			7.978E-11	5.23	Ptom.003G.00826; Ptom.003G.00827
QAO-DCA	Chr16_7876321			2.581E-07	7.71	
QAO-DCA	Chr15_8390069			3.136E-07	4.12	
QAO-DCA	Chr17_8278600			3.257E-07	4.42	
1-O-CGA	Chr1_4984308	1.39	-0.75	1.218E-11	22.96	
1-O-CGA	Chr1_19206143			4.14E-10	10.59	
1-O-CGA	Chr1_19976404			5.265E-08	15.93	Ptom.001G.01935
1-O-CGA	Chr11_3523861	0.86	-1.53	2.549E-07	25.61	Ptom.011G.00299
1-O-CGA	Chr11_3820242	1.22	-1.34	1.942E-07	30.74	
1-O-CGA	Chr11_3828779			1.639E-07	19.47	
1-O-CGA	Chr11_7662950			3.284E-07	13.65	Ptom.011G.00539
1-O-CGA	Chr13_4968097			4.064E-07	17.51	
1-O-CGA	Chr15_8263018	1.42	-0.65	2.386E-08	16.89	
1-O-CGA	Chr17_1450651			8.515E-08	12.75	Ptom.017G.00129
1-O-CGA	Chr17_2408777	1.48	-0.10	1.364E-07	29.24	
1-O-CGA	Chr12_8276505			2.399E-07	21.77	Ptom.012G.00675; Ptom.012G.00674
1-O-CGA	Chr19_17549713			2.55E-07	22.74	
3-O-CGA	Chr18_9299320			1.076E-08	25.15	Ptom.018G.00976
3-O-CGA	Chr18_10901244			1.245E-08	24.47	Ptom.018G.01130
3-O-CGA	Chr12_10858542			1.698E-07	23.47	Ptom.012G.00939
3-O-CGA	Chr1_16365189			3.406E-07	17.83	Ptom.001G.01742
3-O-CGA	Chr17_9296943			3.81E-07	18.68	Ptom.017G.00821; Ptom.017G.00822; Ptom.017G.00823

3-O-CGA	Chr1_11984361			4.072E-07	21.63	Ptom.001G.01332; Ptom.001G.01331
3-O-CGA	Chr18_4230194			1.202E-07	17.16	
3-O-CGA	Chr16_14739883			1.795E-07	18.48	Ptom.016G.01281
3-O-CGA	Chr16_9657067			1.962E-07	18.15	Ptom.016G.00813
3-O-CGA	Chr18_11786965			2.381E-07	17.74	
3-O-CGA	Chr3_18929877	1.28	-1.89	9.273E-08	16.58	Ptom.003G.01892
3-O-CGA	Chr9_6689099	0.21	-1.16	1.716E-08	17.25	Ptom.009G.00855
5-O-CGA	Chr17_8284020			4.416E-08	25.6	
5-O-CGA	Chr7_8132523			3.22E-07	23.74	Ptom.007G.00722
5-O-CGA	Chr14_14645708			9.105E-08	22.00	
5-O-CGA	Chr3_15288544			1.058E-08	24.28	Ptom.003G.01721; Ptom.003G.01720
5-O-CGA	Chr5_9538404			1.133E-07	24.70	Ptom.005G.01160
5-O-CGA	Chr2_15062495	1.71	-2.72	1.223E-07	15.64	Ptom.002G.02014; Ptom.002G.02015
5-O-CGA	Chr3_10260712			1.392E-07	20.83	Ptom.003G.01317; Ptom.003G.01316; Ptom.003G.01318
5-O-CGA	Chr3_10716590			1.516E-07	21.55	Ptom.003G.01386; Ptom.003G.01384
5-O-CGA	Chr1_24245403			2.655E-07	20.22	
5-O-CGA	Chr11_16696789			2.917E-07	20.27	
CGAME	Chr18_4830858			6.295E-08	4.80	
CGAME	Chr1_12369941			2.641E-07	7.17	
CGAME	Chr1_17838810			1.579E-07	7.97	Ptom.001G.01819
CGAME	Chr1_22451500	1.51	-1.93	1.607E-07	5.86	
CGAME	Chr5_5683986			3.812E-07	4.36	Ptom.005G.00738; Ptom.005G.00739
CGAME	Chr4_3216942			9.632E-08	8.51	Ptom.004G.00380; Ptom.004G.00381; Ptom.004G.00379
CGAME	Chr4_15129244			5.538E-08	4.95	
CGAME	Chr4_18075347			7.953E-08	5.90	Ptom.004G.01807; Ptom.004G.01808; Ptom.004G.01806
CGAME	Chr3_3114392	1.86	-1.52	1.769E-07	4.30	

CGAME	Chr3_16580246	1.7	-1.16	1.743E-07	10.38	Ptom.003G.01788; Ptom.003G.01787
CGAME	Chr11_7601385			3.544E-07	19.31	
CGAME	Chr11_16995390			3.713E-07	5.39	Ptom.011G.01209; Ptom.011G.01207
CGAME	Chr16_11634773			5.232E-09	7.51	
CGAME	Chr16_15696584			3.447E-07	7.01	
CGAME	Chr15_4814346			3.577E-07	5.92	Ptom.015G.00399
CGAME	Chr17_1763189			6.154E-08	7.75	
CGAME	Chr17_6701163			2.885E-07	7.23	
CGAME	Chr2_681731			1.587E-07	8.44	Ptom.002G.00130; Ptom.002G.00129; Ptom.002G.00128
CGAME	Chr2_19607415	1.41	-2.10	3.721E-08	3.94	
CGAME	Chr14_12515435			8.02E-08	7.68	Ptom.014G.01410; Ptom.014G.01401
CGAME	Chr12_1743181			4.133E-07	5.56	Ptom.012G.00123; Ptom.012G.00124
CGAME	Chr12_11937872			1.035E-07	7.58	Ptom.012G.01090; Ptom.012G.01091
CGAME	Chr19_17549713			2.55E-09	5.75	
3-O-*P*-CGAO-H	Chr6_4922262			1.187E-07	24.32	Ptom.006G.00618
3-O-*P*-CGAO-H	Chr15_5514679	1.60	-0.26	2.884E-07	24.68	
3-O-*P*-CGAO-H	Chr18_4081252			4.077E-07	22.21	Ptom.018G.00389
3-O-*P*-CGAO-H	Chr2_1691138			5.294E-08	24.46	
3-O-*P*-CGAO-H	Chr1_28208151	1.25	-0.27	8.439E-08	23.87	Ptom.001G.02582; Ptom.001G.02583
3-O-*P*-CGAO-H	Chr2_4098153	1.48	-0.42	1.068E-07	23.75	Ptom.002G.00650
3-O-*P*-CGAO-H	Chr14_16894188	0.44	-0.36	1.558E-07	20.70	
3-O-*P*-CGAO-H	Chr7_277432			1.843E-07	21.54	Ptom.007G.00044; Ptom.007G.00045; Ptom.007G.00046
3-O-*P*-CGAO-H	Chr8_14803	1.46	-0.42	2.35E-08	21.69	Ptom.008G.00003; Ptom.008G.00004; Ptom.008G.00002
3-O-*P*-CGAO-H	Chr14_9394173	1.24	-0.10	2.359E-07	21.84	
O-FQA	Chr18_4830858			2.061E-07	4.66	
O-FQA	Chr1_6574176			3.427E-07	4.86	Ptom.001G.00737; Ptom.001G.00738

O-FQA	Chr1_7881079			1.576E-07	16.28	Ptom.001G.00888; Ptom.001G.00887
O-FQA	Chr1_22451500	1.41	-1.88	6.924E-08	6.71	
O-FQA	Chr1_35890321			1.864E-07	14.89	
O-FQA	Chr1_40520390	1.24	-2.09	4.489E-08	21.26	
O-FQA	Chr4_3216942			1.844E-07	8.37	Ptom.004G.00380; Ptom.004G.00381; Ptom.004G.00379
O-FQA	Chr4_15129244			3.742E-08	5.56	
O-FQA	Chr4_18075347			1.444E-07	3.01	Ptom.004G.01807; Ptom.004G.01808; Ptom.004G.01806
O-FQA	Chr3_3114392	1.78	-1.46	1.041E-07	4.93	
O-FQA	Chr3_16580246	1.78	-1.45	9.089E-08	12.22	Ptom.003G.01788; Ptom.003G.01787
O-FQA	Chr11_15926731			3.309E-07	11.38	Ptom.011G.01119
O-FQA	Chr16_11634773			6.418E-08	6.44	
O-FQA	Chr16_15696584			2.511E-07	7.52	
O-FQA	Chr13_3605392			1.603E-07	10.15	Ptom.013G.00337
O-FQA	Chr17_1763189			1.841E-07	7.40	
O-FQA	Chr2_19607415	1.35	-2.01	1.604E-07	3.74	
O-FQA	Chr14_2259822			2.579E-07	9.10	Ptom.014G.00243
O-FQA	Chr14_12515435			4.854E-08	8.28	Ptom.014G.01410; Ptom.014G.01401
O-FQA	Chr19_8739474	0.67	0.23	3.992E-07	29.91	
1-O-FQA	Chr6_449913			3.812E-08	8.62	
1-O-FQA	Chr6_6311435			3.602E-07	6.75	Ptom.006G.00814; Ptom.006G.00813; Ptom.006G.00812
1-O-FQA	Chr6_13797957			2.011E-07	8.86	
1-O-FQA	Chr9_4005236			2.197E-08	10.97	Ptom.009G.00451; Ptom.009G.00450
1-O-FQA	Chr9_5152868	0.97	0.06	2.239E-07	7.10	Ptom.009G.00611; Ptom.009G.00614; Ptom.009G.00613
1-O-FQA	Chr9_6689099	1.31	-0.26	9.048E-09	16.14	Ptom.009G.00854; Ptom.009G.00855
1-O-FQA	Chr10_2397154	1.33	-0.28	3.216E-08	12.01	Ptom.010G.00370
1-O-FQA	Chr10_10736147	1.31	-0.27	4.025E-08	11.85	Ptom.010G.01537

1-O-FQA	Chr10_16612495			2.858E-08	12.12	Ptom.010G.02193; Ptom.010G.02192
1-O-FQA	Chr8_13727643			5.82E-08	11.78	
1-O-FQA	Chr18_8834360			4.049E-07	8.97	
1-O-FQA	Chr18_13834860	1.37	-0.39	2.846E-07	12.58	Ptom.018G.01372
1-O-FQA	Chr1_11197087			1.168E-07	9.13	Ptom.001G.01246; Ptom.001G.01244
1-O-FQA	Chr1_11287003			1.44E-07	7.31	Ptom.001G.01255
1-O-FQA	Chr1_13344889	1.10	-0.05	1.408E-07	11.2	
1-O-FQA	Chr1_14475749	1.21	-0.17	1.915E-07	10.56	Ptom.001G.01590
1-O-FQA	Chr1_15576072			7.471E-08	9.16	
1-O-FQA	Chr1_20210133	1.26	-0.22	2.45E-07	10.42	
1-O-FQA	Chr1_22396676	1.39	-0.44	2.286E-07	20.6	
1-O-FQA	Chr1_24738065			1.658E-07	7.23	Ptom.001G.02201
1-O-FQA	Chr1_30223620	1.09	-0.05	1.483E-08	10.27	Ptom.001G.02772; Ptom.001G.02773
1-O-FQA	Chr1_37656451			3.785E-07	12.64	Ptom.001G.03385
1-O-FQA	Chr5_1618046			2.896E-09	23.45	Ptom.005G.00208
1-O-FQA	Chr5_4855111			7.116E-08	15.97	Ptom.005G.00635
1-O-FQA	Chr5_7480810			3.785E-07	12.64	Ptom.005G.00920
1-O-FQA	Chr5_12872547			2.06E-07	8.69	
1-O-FQA	Chr4_3592879			4.117E-07	9.05	Ptom.004G.00425; Ptom.004G.00426
1-O-FQA	Chr4_6592109			3.977E-07	6.96	
1-O-FQA	Chr4_8735186			1.07E-07	15.30	
1-O-FQA	Chr4_13771991			2.815E-07	24.8	Ptom.004G.01360; Ptom.004G.01359
1-O-FQA	Chr4_14524766			3.826E-07	7.86	Ptom.004G.01445; Ptom.004G.01447
1-O-FQA	Chr3_10070756	1.45	-0.34	2.744E-07	11.42	Ptom.003G.01299; Ptom.003G.01300
1-O-FQA	Chr3_10337173			2.283E-08	10.06	Ptom.003G.01327
1-O-FQA	Chr3_10700761			6.255E-08	8.91	Ptom.003G.01383; Ptom.003G.01384

1-O-FQA	Chr3_16012322			1.54E-07	7.87	
1-O-FQA	Chr3_19433652			9.785E-08	10.01	Ptom.003G.01920; Ptom.003G.01919; Ptom.003G.01921
1-O-FQA	Chr11_1334024			4.719E-09	9.21	Ptom.011G.00090
1-O-FQA	Chr11_11854835			1.798E-08	12.18	Ptom.011G.00741
1-O-FQA	Chr16_330787			5.237E-09	8.93	
1-O-FQA	Chr16_367595			1.933E-07	5.39	Ptom.016G.00026
1-O-FQA	Chr16_10105432			1.418E-07	10.48	Ptom.016G.00845
1-O-FQA	Chr16_14670950			3.689E-07	11.52	Ptom.016G.01280
1-O-FQA	Chr13_607846			1.797E-07	13.94	
1-O-FQA	Chr13_4399097			1.503E-08	10.28	Ptom.013G.00423; Ptom.013G.00424
1-O-FQA	Chr13_14506079			3.5E-07	11.36	Ptom.013G.01383; Ptom.013G.01382
1-O-FQA	Chr15_1105976			4.245E-08	10.39	
1-O-FQA	Chr15_5213074	1.13	-0.07	2.061E-07	9.18	Ptom.015G.00423
1-O-FQA	Chr15_7290052	1.42	-0.37	1.341E-07	11.61	Ptom.015G.00551
1-O-FQA	Chr15_10930191	1.45	-0.34	2.744E-07	11.42	Ptom.015G.00955; Ptom.015G.00953
1-O-FQA	Chr17_1789694	1.09	-0.04	2.137E-07	8.99	
1-O-FQA	Chr2_2415313			2.549E-07	15.48	Ptom.002G.00389; Ptom.002G.00388
1-O-FQA	Chr2_4470015	1.07	-0.02	9.469E-08	8.96	Ptom.002G.00700
1-O-FQA	Chr2_14959480			3.151E-07	10.78	Ptom.002G.02006; Ptom.002G.02005
1-O-FQA	Chr2_22731613			1.005E-07	12.27	
1-O-FQA	Chr7_4035333			3.032E-07	5.94	Ptom.007G.00431
1-O-FQA	Chr7_10579498			9.671E-08	9.17	
1-O-FQA	Chr7_11567450			6.741E-08	5.21	Ptom.007G.01183; Ptom.007G.01185
1-O-FQA	Chr14_12471753			3.56E-07	14.33	
1-O-FQA	Chr12_6859934			8.76E-08	9.00	
1-O-FQA	Chr12_12339429			3.297E-08	7.54	

3-O-FQA	Chr6_179280			7.931E-08	21.43	Ptom.006G.00027; Ptom.006G.00026
3-O-FQA	Chr6_4371728			1.473E-09	13.55	Ptom.006G.00558; Ptom.006G.00559
3-O-FQA	Chr9_6657632	0.51	0.39	2.115E-07	9.94	
3-O-FQA	Chr8_11239562			3.86E-10	16.71	Ptom.008G.01598
3-O-FQA	Chr18_14503358			1.517E-08	9.73	
3-O-FQA	Chr1_26484725			1.175E-14	18.9	Ptom.001G.02390
3-O-FQA	Chr1_28238883			9.784E-09	8.63	Ptom.001G.02585; Ptom.001G.02587
3-O-FQA	Chr5_2108771			3.661E-10	7.06	Ptom.005G.00288; Ptom.005G.00286<br>Ptom.005G.00289; Ptom.005G.00287
3-O-FQA	Chr4_12936442			4.079E-07	12.08	Ptom.004G.01287; Ptom.004G.01288
3-O-FQA	Chr11_12089470			1.879E-07	12.10	
3-O-FQA	Chr17_284009	0.64	-1.47	4.055E-09	14.58	
3-O-FQA	Chr17_2938328			2.613E-07	5.97	Ptom.017G.00288
3-O-FQA	Chr17_7261340			2.936E-07	5.74	Ptom.017G.00640; Ptom.017G.00638; Ptom.017G.00639
3-O-FQA	Chr7_12553618			7.059E-08	7.26	Ptom.007G.01273
3-O-FQA	Chr14_1505062			6.806E-08	11.11	Ptom.014G.00193; Ptom.014G.00194

Table S5. mGWAS and eQTN-associated genes located in the selective sweep region of the genome

Chr	Start	End	Gene_ID	Gene_position
Chr1	11284001	11286000	Ptom.001G.01255	G
Chr11	3522001	3524000	Ptom.011G.00299	G
Chr11	7652001	7656000	Ptom.011G.00539	G
Chr11	16992001	16994000	Ptom.011G.01207	G
Chr12	1738001	1740000	Ptom.012G.00123	G
Chr14	2244001	2246000	Ptom.014G.00243	G
Chr14	10428001	10430000	Ptom.014G.01270	G
Chr14	12476001	12492000	Ptom.014G.01410	G
Chr14	12504001	12516000	Ptom.014G.01401	G
Chr16	6974001	6976000	Ptom.016G.00646	G
Chr17	4010001	4012000	Ptom.017G.00416	G
Chr3	18934001	18936000	Ptom.003G.01892	G
Chr5	6222001	6224000	Ptom.005G.00813	G
Chr6	4368001	4370000	Ptom.006G.00558	G
Chr7	8122001	8124000	Ptom.007G.00722	G
Chr7	12824001	12826000	Ptom.007G.01300	G
Chr9	6692001	6694000	Ptom.009G.00855	G
Chr1	14378001	14380000	Ptom.001G.01582	Q
Chr1	34792001	34794000	Ptom.001G.03197	Q
Chr1	34900001	34908000	Ptom.001G.03207	Q
Chr1	44282001	44284000	Ptom.001G.03842	Q
Chr10	1898001	1900000	Ptom.010G.00305; Ptom.010G.00306	Q

Chr10	9786001	9788000	Ptom.010G.01424	Q
Chr10	13814001	13816000	Ptom.010G.01912	Q
Chr11	3018001	3020000	Ptom.011G.00251	Q
Chr11	4952001	4966000	Ptom.011G.00360	Q
Chr11	9466001	9468000	Ptom.011G.00613	Q
Chr11	11750001	11752000	Ptom.011G.00734	Q
Chr12	780001	784000	Ptom.012G.00064	Q
Chr12	3040001	3042000	Ptom.012G.00249	Q
Chr12	4262001	4264000	Ptom.012G.00356	Q
Chr12	5988001	5990000	Ptom.012G.00524	Q
Chr12	7896001	7898000	Ptom.012G.00643	Q
Chr12	11850001	11854000	Ptom.012G.01081	Q
Chr12	13240001	13242000	Ptom.012G.01260	Q
Chr13	1442001	1448000	Ptom.013G.00137	Q
Chr13	2178001	2180000	Ptom.013G.00184	Q
Chr13	4134001	4142000	Ptom.013G.00395	Q
Chr13	4142001	4144000	Ptom.013G.00396	Q
Chr13	4152001	4154000	Ptom.013G.00397	Q
Chr14	6192001	6200000	Ptom.014G.00756	Q
Chr14	6318001	6320000	Ptom.014G.00771	Q
Chr14	6446001	6452000	Ptom.014G.00790	Q
Chr14	6454001	6456000	Ptom.014G.00791	Q
Chr14	8856001	8858000	Ptom.014G.01129	Q
Chr14	15242001	15254000	Ptom.014G.01501	Q
Chr14	16012001	16014000	Ptom.014G.01529	Q
Chr15	1790001	1792000	Ptom.015G.00188	Q

Chr15	3662001	3664000	Ptom.015G.00305; Ptom.015G.00304	Q
Chr15	5834001	5836000	Ptom.015G.00446	Q
Chr15	6834001	6836000	Ptom.015G.00519	Q
Chr15	7484001	7486000	Ptom.015G.00576	Q
Chr15	8006001	8008000	Ptom.015G.00620	Q
Chr16	516001	518000	Ptom.016G.00038	Q
Chr16	4812001	4814000	Ptom.016G.00483; Ptom.016G.00482	Q
Chr16	6140001	6144000	Ptom.016G.00614	Q
Chr16	6558001	6560000	Ptom.016G.00631	Q
Chr17	764001	766000	Ptom.017G.00043	Q
Chr17	2946001	2952000	Ptom.017G.00289	Q
Chr17	5392001	5396000	Ptom.017G.00521	Q
Chr17	5638001	5640000	Ptom.017G.00528	Q
Chr17	8044001	8046000	Ptom.017G.00720	Q
Chr17	10846001	10850000	Ptom.017G.00909	Q
Chr17	12416001	12418000	Ptom.017G.01009	Q
Chr19	9402001	9404000	Ptom.019G.00548	Q
Chr2	2442001	2444000	Ptom.002G.00396	Q
Chr2	10958001	10964000	Ptom.002G.01571	Q
Chr2	15906001	15914000	Ptom.002G.02092	Q
Chr2	18418001	18420000	Ptom.002G.02253	Q
Chr2	21666001	21668000	Ptom.002G.02503	Q
Chr2	21762001	21768000	Ptom.002G.02508	Q
Chr2	22072001	22076000	Ptom.002G.02528	Q
Chr3	13888001	13890000	Ptom.003G.01633	Q
Chr3	15772001	15774000	Ptom.003G.01749	Q

Chr3	16066001	16068000	Ptom.003G.01760	Q
Chr3	18934001	18936000	Ptom.003G.01892	Q
Chr4	1370001	1376000	Ptom.004G.00158	Q
Chr4	1440001	1442000	Ptom.004G.00167	Q
Chr4	3222001	3224000	Ptom.004G.00383	Q
Chr4	18410001	18412000	Ptom.004G.01837	Q
Chr5	16494001	16496000	Ptom.005G.01695	Q
Chr6	818001	820000	Ptom.006G.00122	Q
Chr6	2600001	2602000	Ptom.006G.00325	Q
Chr6	4102001	4110000	Ptom.006G.00533; Ptom.006G.00532; Ptom.006G.00534	Q
Chr6	5994001	5996000	Ptom.006G.00780	Q
Chr6	9556001	9558000	Ptom.006G.01155	Q
Chr6	15148001	15150000	Ptom.006G.01595	Q
Chr6	18294001	18296000	Ptom.006G.01928	Q
Chr6	22118001	22120000	Ptom.006G.02487	Q
Chr6	23142001	23148000	Ptom.006G.02629	Q
Chr7	8800001	8802000	Ptom.007G.00797	Q
Chr7	9226001	9308000	Ptom.007G.00873	Q
Chr7	9876001	9878000	Ptom.007G.00941	Q
Chr8	7382001	7384000	Ptom.008G.01100	Q
Chr8	9494001	9496000	Ptom.008G.01377	Q
Chr9	6692001	6694000	Ptom.009G.00855	Q
Chr9	8530001	8532000	Ptom.009G.01181	Q

G and Q represent the associated genes in mGWAS and eQTN mapping, respectively.

Table S6. Gene Ontology (GO) enrichment analysis of all genes detected by mGWAS and eQTN mapping (FDR ≤ 0.05)

Class	Name	ID	Level	*P*-value[a]	Score	HGC_SS[b]	HGC_BG[c]	AGC_SS[d]	AGC_BG[e]	*p*-value[f]
Molecular function	gated channel activity	GO:0022836	6	7.94E-04	3.62	9	71	539	15404	0.16
Molecular function	antioxidant activity	GO:0016209	2	4.17E-03	2.54	11	124	539	15404	0.27
Molecular function	peroxidase activity	GO:0004601	5	6.26E-03	2.53	10	113	539	15404	0.35
Molecular function	oxidoreductase activity, acting on peroxide as acceptor	GO:0016684	4	6.66E-03	2.51	10	114	539	15404	0.33
Molecular function	ion channel activity	GO:0005216	6	2.06E-02	2.22	9	116	539	15404	0.74
Molecular function	NADP binding	GO:0050661	6	2.36E-02	3.04	5	47	539	15404	0.77
Molecular function	voltage-gated ion channel activity	GO:0005244	8	2.77E-02	2.92	5	49	539	15404	0.68
Molecular function	voltage-gated channel activity	GO:0022832	7	2.77E-02	2.92	5	49	539	15404	0.68
Molecular function	catalytic activity, acting on a protein	GO:0140096	3	3.27E-02	1.20	95	2268	539	15404	0.54
Molecular function	hydrolase activity, acting on glycosyl bonds	GO:0016798	4	3.69E-02	1.51	22	417	539	15404	0.47
Cellular component	ribosomal subunit	GO:0044391	4	3.47E-03	4.76	5	30	156	4452	0.62
Biological process	system development	GO:0048731	4	4.15E-04	3.61	10	76	415	11375	0.29
Biological process	sister chromatid segregation	GO:0000819	7	1.28E-03	4.17	7	46	415	11375	0.45
Biological process	mitotic cell cycle	GO:0000278	4	1.42E-03	3.65	8	60	415	11375	0.33

Biological process	nuclear chromosome segregation	GO:0098813	4	2.65E-03	3.69	7	52	415	11375	0.46
Biological process	mitotic sister chromatid segregation	GO:0000070	8	3.05E-03	4.11	6	40	415	11375	0.43
Biological process	mitotic nuclear division	GO:0140014	8	3.05E-03	4.11	6	40	415	11375	0.43
Biological process	mitotic cell cycle process	GO:1903047	4	3.29E-03	3.55	7	54	415	11375	0.33
Biological process	multicellular organism development	GO:0007275	4	3.39E-03	2.47	12	133	415	11375	0.30
Biological process	chromosome segregation	GO:0007059	3	3.66E-03	3.49	7	55	415	11375	0.26
Biological process	isoprenoid biosynthetic process	GO:0008299	7	3.92E-03	3.92	6	42	415	11375	0.18
Biological process	isoprenoid metabolic process	GO:0006720	6	3.92E-03	3.92	6	42	415	11375	0.18
Biological process	electron transport chain	GO:0022900	5	5.63E-03	4.28	5	32	415	11375	0.23
Biological process	response to oxidative stress	GO:0006979	4	8.21E-03	2.43	10	113	415	11375	0.29
Biological process	organelle fission	GO:0048285	6	9.91E-03	2.67	8	82	415	11375	0.19
Biological process	anatomical structure development	GO:0048856	3	2.66E-02	1.88	12	175	415	11375	0.27
Biological process	regulation of cell cycle process	GO:0010564	6	2.75E-02	2.92	5	47	415	11375	0.27
Biological process	cell cycle process	GO:0022402	3	2.83E-02	2.00	10	137	415	11375	0.28
Biological process	nuclear division	GO:0000280	7	3.07E-02	2.53	6	65	415	11375	0.30
Biological process	cell cycle	GO:0007049	3	3.46E-02	1.81	12	182	415	11375	0.32

Biological process	multicellular organismal process	GO:0032501	2	4.69E-02	1.68	13	212	415	11375	0.33

[a]Significance level

[b]Hits genes counts in selected set

[c]Hits genes counts in background

[d]All genes counts in selected set

[e]All genes counts in background

[f]Corrected *p*-value (BH method)

Table S7. Details of the association analysis of candidate genes and target traits in *P. tomentosa*

Gene	Trait	SNP/InDel_ID	SNP/InDel allele	*P*-value	*Q*-value	R^2 (%)
RPL3B	QA	Chr3_18929594	T/A	5.03E-04	7.38E-04	10.08
RPL3B	QA	Chr3_18929877	A/C	5.16E-06	3.78E-05	10.49
RPL3B	QA	Chr3_18929881	A/T	5.45E-05	2.00E-04	9.03
RPL3B	QA	Chr3_18929894	G/T	8.37E-04	8.37E-04	5.57
RPL3B	QA	Chr3_18933578	A/G	1.08E-04	2.37E-04	15.15
RPL3B	QA	Chr3_18936940	G/A	3.63E-04	5.70E-04	13.39
RPL3B	QA	Chr3_18936945	G/A	5.68E-04	7.81E-04	12.29
RPL3B	QA	Chr3_18929839	T/TAA	8.17E-05	2.00E-04	18.78
RPL3B	QA	Chr3_18930011	A/AAATACAG	7.32E-04	8.05E-04	21.53
RPL3B	CGA	Chr3_18929873	G/T	2.51E-04	4.60E-04	12.03
RPL3B	CGA	Chr3_18929877	A/C	1.24E-05	6.82E-05	16.58
RPL3B	CGA	Chr3_18936994	C/T	7.50E-04	7.86E-04	10.94
RPL3B	CGA	Chr3_18929839	T/TAA	6.58E-04	7.62E-04	14.78
ERF109	1-O-FQA	Chr9_6689060	C/G	5.21E-05	2.29E-04	14.42
ERF109	1-O-FQA	Chr9_6689099	T/C	5.78E-07	1.27E-05	16.14
ERF109	1-O-FQA	Chr9_6689130	G/A	6.96E-05	1.91E-04	12.82
ERF109	1-O-FQA	Chr9_6689154	G/T	1.78E-04	3.57E-04	11.88
ERF109	1-O-FQA	Chr9_6689118	GA/G	5.77E-05	1.81E-04	24.57
ERF109	CGA	Chr9_6688982	G/T	6.23E-04	8.06E-04	9.42
ERF109	CGA	Chr9_6689099	T/C	2.39E-06	2.63E-05	17.25
ERF109	CGA	Chr9_6689154	G/T	6.56E-04	8.02E-04	13.83
ERF109	CGA	Chr9_6689118	GA/G	2.57E-04	4.35E-04	22.37

Table S8. Correlation between causal genes expression levels and metabolite abundance

	RPL3B		*MATE1*		*PR1*		*ERF109*		*SIB1*		*DUF5086*	
	SC	*P*-value	SC	*P*-value	SC	*P*-value	SC	*P*-value	SC	*P*-value	SC	*P*-value
RPL3B	1		-0.495**	3.42E-07	0.373**	1.62E-04	-0.154	1.25E-01	-0.047	6.43E-01	0.204*	4.16E-02
MATE1	-0.495**	3.42E-07	1		-0.256*	1.72E-02	0.066	5.13E-01	-0.099	3.29E-01	-0.022	8.29E-01
PR1	0.373**	1.62E-04	-0.256*	1.72E-02	1		0.341**	5.97E-04	0.031	7.64E-01	-0.180	7.58E-02
ERF109	-0.154	1.25E-01	0.066	5.13E-01	0.341**	5.97E-04	1		0.537**	4.32E-09	0.308**	2.33E-03
SIB1	-0.047	6.43E-01	-0.099	3.29E-01	0.031	7.64E-01	0.537**	4.32E-09	1		-0.122	2.25E-01
DUF5086	0.204*	4.16E-02	-0.022	8.29E-01	-0.180	7.58E-02	0.308**	2.33E-03	-0.120	2.25E-01	1	
QA	-0.618**	1.73E-11	0.086	3.94E-01	0.042	6.81E-01	0.128	2.03E-01	0.195	5.16E-02	0.144	1.52E-01
EQA	0.090	3.76E-01	-0.226*	2.44E-02	0.054	6.01E-01	0.014	8.93E-01	0.038	7.09E-01	-0.449**	3.21E-06
QAO-DCA	0.134	1.83E-01	0.115	2.56E-01	-0.179	7.85E-02	-0.157	1.18E-01	-0.029	7.77E-01	0.157	1.18E-01
1-O-CGA	-0.285**	4.25E-03	0.101	3.19E-01	0.046	6.54E-01	0.278**	4.99E-03	0.108	2.89E-01	-0.085	4.04E-01
3-O-CGA	-0.639**	1.28E-12	0.175	8.23E-02	0.047	6.45E-01	0.601**	1.53E-11	0.120	2.35E-01	0.039	7.00E-01
5-O-CGA	0.041	6.89E-01	0.112	2.71E-01	0.174	8.96E-02	0.101	3.24E-01	0.040	6.93E-01	0.218*	3.14E-02
CGAME	0.033	7.47E-01	-0.257**	9.91E-03	0.045	6.61E-01	-0.140	1.63E-01	0.083	4.09E-01	-0.395**	4.66E-05
3-O-*P*-CGAO-H	0.063	5.34E-01	-0.195	5.14E-02	-0.077	4.54E-01	0.038	7.07E-01	0.031	7.61E-01	-0.178	7.66E-02
O-FQA	0.040	6.92E-01	-0.250*	1.25E-02	-0.135	1.36E-01	-0.162	1.09E-01	0.076	4.56E-01	-.386**	8.08E-05
1-O-FQA	-0.028	7.80E-01	-0.074	4.63E-01	-0.413**	1.25E-05	-0.569**	9.51E-10	-0.055	5.88E-01	-.297**	2.69E-03
3-O-FQA	-0.301**	2.50E-03	0.074	4.66E-01	0.025	8.07E-01	-0.009	9.26E-01	0.040	6.94E-01	-0.064	5.28E-01

* and ** represent significant correlation at $P < 0.05$ and $P < 0.01$, respectively.

SC represent spearman correlation.
